# Acceptance and Commitment Therapy for Pediatric Chronic Pain: Theory and Application

**DOI:** 10.3390/children4020010

**Published:** 2017-01-30

**Authors:** Melissa Pielech, Kevin E. Vowles, Rikard Wicksell

**Affiliations:** 1 Department of Psychology, University of New Mexico, Albuquerque, NM 87131, USA; 2 Department of Clinical Neuroscience, Karolinska Institutet, SE-171 76 Stockholm, Sweden; rikard.wicksell@karolinska.se

**Keywords:** Acceptance and Commitment Therapy, ACT, children, adolescents, pediatric, chronic pain, pain acceptance

## Abstract

Acceptance and Commitment Therapy (ACT) is a third wave behavior therapy approach which aims to increase engagement in activities that bring meaning, vitality, and value to the lives of individuals experiencing persistent pain, discomfort, or distress. This goal is particularly relevant when these aversive experiences cannot be effectively avoided or when avoidance efforts risk their exacerbation, all of which may be common experiences in children and adolescents with chronic pain conditions. The primary aim of the present paper is to review and summarize the extant literature on the application, utility, and evidence for using ACT with pediatric chronic pain populations by: (1) defining the theoretical assumptions of the ACT model; (2) summarizing research study findings and relevant measures from the published literature; and (3) critically discussing the strengths, limitations and areas in need of further development.

## 1. Introduction

A significant percentage of young people experience chronic pain, generally defined as pain that persists for three months or longer [1]. A subset of these patients report marked deficits in healthy functioning [2] and commonly experience comorbid mental health difficulties that can persist into adulthood [3]. Treatment efforts for chronic pain often highlight the primacy of pain reduction or elimination. Such efforts to minimize current pain and avoid it in the future are perfectly natural. In acute cases, efficient pain escape and avoidance behaviors can have genuine adaptive value, as they minimize risk of morbidity and mortality by allowing for efficient detection and response to painful or potentially painful situations [4,5,6].

In the case of chronic pain, however, these perfectly natural responses may not be the most adaptive. In fact, when persistent, avoidance behaviors can be reliably associated with significant disruptions in physical, social, and emotional functioning across the lifespan, often without any corresponding decrease in pain. A prime and well-established example of these findings is the fear-avoidance model, which consistently indicates that more persistent and widespread efforts to avoid pain are associated with worse current and future functioning in both pediatric and adult settings [7,8,9,10,11]. In youth, the role of caregiver responses to pain is also highly relevant, as high fear-avoidance in parents or responses to the child’s pain that reinforce avoidance are related to greater levels of child distress and disability [12,13,14]. Thus, when pain avoidance is a primary goal in youth and their caregivers, there appears to be a heightened risk that pain will be more disruptive in important areas of physical and psychosocial functioning [15,16].

Youth with chronic pain and their family systems are therefore likely in need of treatments that emphasize effective responding to pain, with less reliance on pain control. It is possible that effective responding to chronic pain requires somewhat paradoxical responses to pain. Such paradoxical responses might include, for instance, decreasing pain avoidance attempts, particularly when they are ineffective at avoiding pain over the longer term or when they negatively impact important functioning. Further, effective responding to pain may actually include “approach” behaviors, such as participation in meaningful activities even when pain is present. Importantly, adoption of such strategies requires careful consideration regarding whether one is willing to experience pain in the service of engagement in meaningful activities. Acceptance and Commitment Therapy [17] aims to improve the ability to act in alignment with personal values while in the presence of potentially interfering pain and distress, a response pattern defined as psychological (or behavioral) flexibility. As part of this process, the individual is encouraged to explore and challenge the utility of avoidance, as well as acceptance-oriented strategies, in managing chronic pain. The primary aim of the present paper is to provide a narrative review and summary of the extant literature on the application and utility of ACT specifically for pediatric chronic pain. First, we review the primary goals and theoretical assumptions of the ACT model. Second, we summarize treatment outcomes and relevant measures from the published literature. Finally, we discuss strengths, limitations, and areas in need of development.

### 1.1. The ACT Model

While a full review of the theoretical and philosophical assumptions of the ACT model is beyond the scope of the present review (see Hayes et al. [17] for more information), it is important to highlight a few key points. The first is to emphasize the overarching goal of ACT, which is to increase successful engagement in activities that bring meaning, vitality, and importance to the lives of individuals experiencing persistent pain, discomfort, or distress. This goal is particularly relevant when these aversive experiences cannot be effectively avoided or when avoidance efforts risk their exacerbation, as is often the case with persistent pain. Each of the following conceptual assertions circle back to this overarching goal.

ACT is based on the philosophical positions of both pragmatism and functional contextualism [18]. Its pragmatic goal is “effective action,” meaning it aims to facilitate the effectiveness of behavior in achieving adaptive and functional goals over the longer term. At the level of actual clinical interaction, this goal is described in terms of greater engagement in valued actions. The functional contextual orientation of ACT allows one to define two primary aims: (1) accurate prediction and (2) useful influence on behavior [19]. The pursuit of these aims requires one to attend to the relevant contextual events in any analysis of behavior, including historical events giving rise to the behavior as well as relevant ongoing events in the person’s environment. Practically, ACT seeks to undermine the influence of key current and historical stimuli that contribute to ineffective responses to pain, such as persistent avoidance, and bolster the influence of those that contribute to more effective responses, such as the specification and pursuit of desired valued outcomes. These aims of “accurate prediction” and “useful influence on behavior” are concordant with the operant behavioral roots of both ACT and Cognitive-Behavioral Therapy (CBT; [18,19]).

Much discussion has occurred regarding the similarities and differences amongst ACT and other forms of psychotherapy, principally Cognitive Behavioral Therapy (CBT; for information regarding applications of CBT for pediatric chronic pain, please refer to [20,21]). We suggest that there are two key differences between these approaches. The first is the central focus on the facilitation of values-based actions in ACT. While such a focus is both fully compatible and at times apparent within CBT (e.g., [18,19]), its centrality in ACT is distinctive. The second key difference pertains to working with human language and cognition. In brief, ACT seeks not to directly alter the occurrence of certain instances of human cognition (e.g., catastrophic thinking) and sensation (e.g., pain intensity), but to increase the repertoire of responses to these cognitions and sensations, as well as the flexible use of them, to facilitate actions more in line with valued activities over the longer term.

The experimental pain literature provides several examples regarding the utility of altered responding to human cognition and sensation in adults. One of the earliest experimental trials of acceptance involved an acute pain induction task, where participants were asked to submerge a hand into an ice water bath and were randomized to a pain control (e.g., keep your pain under control; don’t let it increase) or pain acceptance (e.g., let your pain be; don’t let it control your responses) set of instructions [22]. Results indicated that tolerance time for participants in the latter condition were significantly longer than the former. Importantly, participants in the pain acceptance condition viewed pain and thoughts about pain as less influential than those in the pain control condition. This pattern of results has been replicated several times in studies with experimentally induced pain and healthy controls [23,24,25,26,27], as well as in adults with low back pain [28]. Furthermore, laboratory studies suggest that the inclusion of values in experimental pain settings is important. For example, when a pain task is paired with a personally important reason for experiencing the pain, tolerance times tend to be increase and the experience of pain is viewed as a less important determinant of behavior [27,29].

The clinical model of ACT specifies several related treatment processes, each of which is intended to help facilitate more effective responding to difficult or aversive experiences. For example, the central overarching process has been termed “psychological flexibility,” which can be simply defined as effectively and flexibly responding to aversives such that engagement in important areas of living is maintained at a level that is sufficient for the needs of the individual [17,30]. Thus, an instance of psychological flexibility in an adolescent with chronic pain may be maintenance of social and scholastic engagement even with the ongoing experience of chronic pain and including times when pain is low as well as when it is high (see Wicksell et al. [31], summarized below, for description of a case example).

Underlying this overarching process of psychological flexibility are three pairs of “sub” processes [17]. These three pairs include (1) acceptance and defusion; (2) moment-to-moment awareness and a transcendent sense of self; and (3) clarity and committed action in pursuit of valued activities (e.g., see [17] for details, as well as [32] for an empirical evaluation of this model in chronic pain). Acceptance and defusion refer to patterns of responding to pain that involve acknowledgement that pain and suffering are a normal part of life many times and choosing to work with these experiences rather than try to avoid or control them [33,34,35]. Moment-to-moment awareness and a transcendent sense of self refer to aspects of mindfulness that seek to increase consistent, nonjudgmental attention to the present moment, less struggling with present experiences, and the facilitation of a stable sense of self as a person having experiences, rather then an unstable sense of self as a person who is defined by the experiences themselves (see [36,37]). Finally, clarity and committed action in relation to values includes a definition of useful, valued directions to help guide behavior while in the midst of difficult circumstances, as well as a flexible commitment to these values such that behavior can be adjusted over time to facilitate consistent movement towards them [38].

Thus, treatment success in ACT for pediatric chronic pain could be defined as the occurrence of effective responding to the natural variations in pain intensity that occur, such that personal needs and goals are being met or progressed. Furthermore, the model assumes that such treatment success is most likely to occur when one is (1) aware of pain when it is occurring, but is not consumed by that experience to the exclusion of other things happening; (2) aware of present experiences as they are occurring and able to let these experiences come and go; and (3) clear on valued areas and engaged in a flexibly persistent pattern of behaviors to pursue these areas. Overall, the relevance of these processes in adult chronic pain is reasonably well-established in that measures of these processes are reliably and significantly related with pain-related distress, disability, and healthcare use [39,40,41].

In the case of pediatric pain treatment specifically, this model applies to both caregiver and child. Much like caregiver responses are important in the fear-avoidance model, greater caregiver acceptance of pain and discomfort is associated with less restricted functioning in children [42,43]. Thus, it can be hypothesized that parental support of the child’s engagement in valued activities in the presence of pain via operant approaches may be helpful to the child’s overall success in adapting effectively to a chronic condition.

### 1.2. Developmental Considerations

ACT has been successfully adapted and implemented for use with a wide range of pediatric populations including youth with chronic pain (e.g., [44]), cystic fibrosis [45], and anxiety disorders [46], as well as medical conditions that involve pain as a primary symptom [47]. For a review of the application of ACT more broadly with pediatric populations with physical health conditions, please refer to [48]. Given the inherent level of abstraction in some ACT concepts, developmentally sensitive modifications in the language and delivery of treatment may be necessary. Adaptations should be made based on clinical judgment and awareness of the patient’s level of cognitive, social, and psychological functioning, as well as abstraction abilities (refer to [49] for a discussion of adapting ACT interventions for adolescent populations). Briefly, adaptations may include age-appropriate simplification of language of complex concepts, such as referring to distressing, pain-related thoughts as coming from a “pain monster” whose advice may serve to restrict functioning and effective engagement in valued activities [31]. Concrete strategies can be used for teaching abstract concepts, like facilitation of values identification by using a heart shaped box that is filled with slips of paper describing patient values or teaching mindfulness via a walking exercise [50]. There are also numerous ACT metaphors that are developmentally relevant for use with adolescents, though it is recommended that use of metaphors be chosen with consideration to the patient’s social context and interests. It can also be helpful to reiterate and repeat important topics to reinforce understanding. Additionally, the importance of family factors and inclusion of parents/caregivers in pediatric pain treatment is of clear importance [14,31,51]. From an ACT perspective, more traditional behavioral strategies such as contingency management may favorably be combined with interventions aimed at improving the parent’s ability to self-manage distress, which otherwise may interfere with effective coaching [33].

## 2. The Evidence-Base for ACT with Youth with Chronic Pain

### 2.1. Search Criteria

Relevant treatment outcome papers using ACT published through December 2016 were identified through searches on PubMed and PsychInfo using search terms such as ‘Acceptance and Commitment Therapy’ or ‘ACT’ and ‘children,’ ‘adolescents,’ ‘youth,’ or ‘pediatric’ and ‘chronic pain.’ The evidence base for using ACT with youth for chronic pain is modest, and includes one case study, one case series, two randomized controlled trials, and three prospective cohort studies. Each of these studies is reviewed below. In addition, a fourth cohort study is also reviewed. While the study described a CBT-based intervention, mediation analyses were done using a measure of pain acceptance, thus the outcomes seem relevant to include.

### 2.2. Case Study and Case Series

The first study in the field of ACT for pediatric chronic pain is a case study by Wicksell et al. [33] that facilitated development of ACT treatments for youth with chronic pain and was helpful in distinguishing between ACT and CBT in the context of chronic pain rehabilitation. The patient was a 14-year-old female, who had been experiencing persistent pain for three years. Her array of symptoms were conceptualized as “musculoskeletal pain syndrome” and included generalized joint and body pain, persistent headaches, and features of panic attacks with subsequent school absence, social isolation from friends, and withdrawal from valued activities. Treatment was rehabilitative in nature and primarily consisted of exposure to stimuli related to pain and distress, including avoided activities and places, as well as significant values clarification exercises. Treatment comprised a total of 13 sessions over a six-month period, with three of the 13 sessions including both patient and parents. At discharge, improvements were noted in functional disability, engagement with valued activities, scholastic involvement, and avoidance of emotions. Importantly, these improvements were sustained during a six-month follow up period.

In 2007, a follow-up case series was published describing findings from an individual ACT-based treatment approach used with 14 adolescents (11 females, mean age = 17 years, standard deviation (SD = 2.1) with chronic pain and high levels of pain-related disability [52]. Primary outcome variables were disability and school attendance. Treatment emphasized exposure to private events and previously avoided activities, values clarification and use of values as guiding principles for engagement in exposure, as well as acceptance of distressing and negative feelings. Treatment length varied from 5–29 weekly sessions and included individual sessions with parents, as needed. Significant improvements were observed in the primary outcomes variables, which were maintained through 3 and 6-month follow-ups. Significant improvements were also observed in levels of pain catastrophizing, pain intensity, and pain interference.

### 2.3. Randomized Controlled Trials (RCT)

Following the case study and pilot case series, Wicksell et al. [44] published the first RCT evaluating the effectiveness of an ACT-based treatment for children and adolescents with chronic pain. ACT was compared to a multidisciplinary treatment (MDT) including amitriptyline medication. The primary components of the 10-week ACT-oriented treatment group were acceptance strategies and exposure, with sessions occurring weekly. A total of 32 youth participated (mean age = 14.8 years, SD = 2.4) with 16 patients randomized to each condition. Patients in the ACT condition improved significantly in multiple domains (e.g., functional disability, health related quality of life) and changes were sustained through follow up, as evidenced by multiple large effect sizes. Patients in the MDT group improved in many domains, as well, but across conditions, patients who received ACT improved significantly more in levels of pain-related fear, overall quality of life, pain intensity, and pain interference. A prolonged treatment period in the MDT condition complicated comparisons at follow up, but results demonstrated the relative utility of ACT in comparison to a multidisciplinary treatment.

In a planned set of post-hoc analyses, Wicksell et al. used data from this same RCT to identify mediators of treatment outcome [41]. Tested mediators were pain intensity, as well as both CBT- and ACT-consistent variables, including self-efficacy, catastrophizing, kinesiophobia, pain-impairment beliefs, and pain reactivity. Only these last two variables, which were argued to be representative of a more flexible and willing pain response style, were shown to be significant mediators of change. Furthermore, in subsequent analyses these same two mediators were independent predictors of outcomes at follow-up for the ACT condition only. Although tentative, the pattern of results suggests that variables consistent with psychological flexibility mediate the effects of ACT-based interventions to improve functioning in patients with chronic debilitating pain.

In a more recent study, Ghomian and Shairi [53] compared an ACT based treatment (*n* = 10) with a control condition (*n* = 10) in children ages 7 to 12 with chronic pain. Details regarding treatment components or the control condition were not provided (Note. the study authors were emailed a request asking for further details regarding treatment and control conditions and no response was received). Data was collected at four time points: pre-treatment, discharge, and 3.5 and 6.5 months post-treatment. Regarding results, patients in the ACT condition reportedly demonstrated significantly greater improvements in functional disability at the end of treatment and through the follow up time points.

### 2.4. Prospective Cohort Studies

Gauntlett-Gilbert et al. [54] evaluated outcomes from a 3-week residential, interdisciplinary ACT-based program (~90 h of treatment) for adolescents (*n* = 98) with chronic pain. The program, comprised of physical conditioning, activity management, and psychology, also included parent involvement in most sessions, with the exception of a four-day period where parents received therapy separately from their child. Data on self-reported functioning (e.g., depressive and anxious symptoms, levels of pain acceptance) and objective physical ability was collected from child and parent across three time points: baseline, three weeks after discharge from treatment, and at a three-month follow up time point. Patients improved across all domains of functioning in a manner that was theoretically consistent with ACT whereby improvements occurred without efforts to control pain or manipulate cognitions. Additionally, improvements in pain-related acceptance were associated with better treatment outcomes, suggesting a key role of acceptance in pediatric pain rehabilitation.

Martin et al. [55] published outcomes of a feasibility trail of ACT for adolescents with chronic pain and Neurofibromatosis type 1 (NF1), an autosomal disorder. The sample included 10 adolescents, who averaged 17 years of age and seven parents, who participated in a two day group intervention provided in a “workshop” format. At a three-month follow-up, significant declines in pain interference were reported by both patients and parents, while patients only reported decreased pain intensity. The authors suggested that the obtained data supported the feasibility of ACT in young people diagnosed with NF1.

#### Prospective Studies with Parents

Recently, Wallace et al. [56] published the first pilot study examining the application of an eight-week, ACT-based group intervention with parents (*n* = 8; 6 completed the study) of youth with chronic pain. The primary treatment target was to increase parental psychological flexibility, specifically for parents to identify areas of ineffective action or stuckness within themselves and their family unit, as well as develop strategies to support pursuit of values-based action. Sessions were delivered once a week, on an outpatient basis, and were 75 min long (session by session content is detailed by the authors in the manuscript [56]). Measures of parent psychological flexibility, responses to child pain symptoms, and levels of pain interference were collected during treatment and at three follow up time points, up to six months post treatment. Overall, parents were highly satisfied with the intervention. Furthermore, parent levels of psychological flexibility increased during treatment, as well as through follow up. During follow up, parent protective responses and adolescent-reported levels of pain interference decreased significantly. The authors hypothesize that the delayed improvements observed in parental responses may indicate a potential mediating effect of psychological flexibility on parental responses.

A final study examined changes in pain-acceptance following CBT for chronic pain. While this study was not strictly an application of ACT, the treatment description suggests that there were some goals that were concordant. For example, the authors of the study noted that participants and their parents were told that the goal of treatment was not decrease pain, but to “increase coping skills, functioning, and quality of life” [34]. Thus, it seemed appropriate to include this study within the present review, particularly because it examined how changes in acceptance over the course of the treatment were related to changes in distress and disability. A total of 112 youth, aged 11–18, participated in the treatment program, which included daily relaxation training, physical therapy, occupational therapy, recreation therapy, family therapy, and psychotherapy groups. Levels of acceptance significantly increased during treatment, while levels of depression, pain catastrophizing, and functional disability significantly decreased. Importantly, changes in acceptance significantly predicted changes in all psychosocial variables and functional disability.

## 3. Measures of ACT Processes in Youth with Chronic Pain and Their Families

Table 1 describes self-report measures for youth and their caregivers that assess constructs relevant to ACT processes and their relation with other psychosocial measures of pain-related functioning. In brief, to date there are four measures that explicitly measure ACT processes in children and adolescents, and three questionnaires developed for use with caregivers either as a proxy report (*n* = 1) or a report of the caregiver’s own experience and behaviors (*n* = 3). Three measures not specifically validated with pediatric chronic pain populations were included due to their utility in assessing ACT processes [57,58,59]. Further research and evaluation on all of the measures is warranted, as the statistical properties have only been preliminarily validated. Notably, however, findings from the published cross-sectional studies with pediatric samples are consistent with similar studies in adults and illustrate that ACT-relevant processes are related to functioning in a manner consistent with the underlying theory of ACT (e.g., greater pain acceptance is associated with better emotional and physical status).

## 4. Strengths, Limitations, and Future Directions

ACT takes a pragmatic and flexible approach to the treatment of pediatric pain. The overarching goal of ACT is to decrease ineffective struggles for control of pain or distressing emotions and increase adaptive responses to pain and facilitate consistent re-engagement with valued activities. Theoretically, the probability of this occurring is assumed to be more likely when one approaches aversive experiences with more openness and fewer struggles for control, and when one is more aware of both one’s personal values and currently available opportunities to engage in valued activities.

Although preliminary, the growing body of evidence regarding ACT for pediatric pain is promising and results from existing studies are consistent, offering some support regarding validity and utility. Notably, and unfortunately, the number of studies on ACT for pediatric pain patients is considerably smaller than the body of research and strong evidence for using ACT for adult chronic pain [65]. Furthermore, beyond a small number of studies, the literature on ACT for children and adolescents suffers from either an absence of or poorly defined control conditions, and small sample sizes. Thus, there is a need for more studies and those of higher quality in terms of study design, evaluation across settings (e.g., outpatient, day hospital, individual vs. group), and further identification and replication of the mechanisms by which improvements in functioning occur during treatment. Formal inclusion of parents in treatment and assessment of parent pain-related functioning is also needed to illuminate and optimize their role in their child’s pain rehabilitation.

At present, there is also a need for precise measurement of ACT treatment processes in youth with chronic pain. The adult chronic pain literature boasts numerous measures of ACT constructs, such as psychological flexibility [66,67], pain acceptance [33], engagement in valued activities [68,69,70], and committed action [38], which could potentially be adapted for use with pediatric populations. While there is one measure of pain acceptance for youth, it is curious that no measure of values or assessments related to other aspects of the model have been developed yet for pediatric chronic pain populations. Given the focus within ACT on improvements in values-based actions, the development of a robust measurement method in young people with chronic pain appears highly important. In particular, valued domains in adults may be different than in youth and these differences may require a carefully considered assessment. Furthermore, clinical experience suggests that certain times in one’s life, for example, adolescence, are a time of personal determination of what is of personal value. For example, there can be consideration and weighing of socially-constructed values (e.g., be popular) in relation to those that are more personal in nature (e.g., be kind to others). The adult literature on values assessment offers little to no guidance on the assessment of “values formation” (for lack of a better term) and this seems a distinct and important opportunity for those that work in pediatric settings. In addition, further specification of the unique and interactive effects of caregiver and child responses to pain within an ACT framework appears to present an important opportunity for further work. Thus, while some aspects of the ACT model may be appropriate to assess via a process of “downward” extension from adults to youth, there may be particular facets of the pediatric setting that are deserving of careful consideration.

There are several additional considerations in relation to the use of ACT in the treatment of pediatric chronic pain. First, there is little guidance on ages that are appropriate or inappropriate for ACT; notably, only two of the ten studies reviewed explicitly made mention of developmental adaptations or considerations taken into their protocol [31,55]. In particular, it is not currently known if there is a minimum age, or minimum set of developmental milestones that must have been met, for successful treatment participation. The authors’ clinical experience suggests somewhere in the 8–10 range may be a lower age limit, but this intuition is in need of empirical examination. Second, there is little guidance on the selection of type of psychological treatment, for example, use of ACT instead of CBT. Given the overlap between approaches, it may be that firm data-based rubrics are unlikely in the near future. Furthermore, given the distinctions between ACT and CBT, as detailed in the first major section of this paper, perhaps ACT is more relevant for use in individuals with significant deficits in values clarity and pursuit of values-based directions, or in those who are so paralyzed by pain and associated cognitions that cognitive methods are unlikely to work in an efficient manner. Third, while caregiver involvement in treatment is important and there is evidence demonstrating that caregiver responses and functioning impact child outcomes, the exact type or duration of such involvement is not clear. For example, is it sufficient to simply have caregivers sit in on some or all treatment sessions, or is it necessary for caregivers to receive treatment specific to their particular needs? Again, clinical experience suggests that the latter is more likely to be of use, but, to our knowledge, there are no data to use for guidance. It seems fair to note that these three issues are important for psychological interventions for pediatric chronic pain writ large and seem necessary to address for the field to move forward to firmer empirical ground.

## 5. Conclusions

To conclude, a primary goal of ACT for pediatric chronic pain is to support youth in healthy functioning and aid them in the process of re-engagement in valued activities so that their focus may shift back to age- appropriate activities, rather than predominantly on pain control or pain avoidance. The theoretical principles of ACT provide a framework for the structure of such treatment. While the extant data is promising, much more work needs to be done to augment the evidence base for the application of ACT with pediatric pain populations and to ensure that clinical practice is consistent with the hypothesized theoretical principles of ACT.

## Figures and Tables

**Table 1:** Measures of Acceptance and Commitment Therapy (ACT) processes in pediatric chronic pain.

Measures	Description	Relations
**Child Specific**		
Chronic Pain Acceptance Questionnaire: Adolescent version (CPAQ-A; [60,61])	20 item self-report measure, adapted from the adult version [33]. Assesses two aspects of pain acceptance: (1) Activity Engagement and (2) Pain Willingness. Response options range from (0) *never true* to (4) *always true*.	Correlated with disability, depression, anxiety, self-efficacy. Not correlated with pain-specific variables (e.g., pain duration).
Child and Adolescent Mindfulness Measure (CAMM; [58]) *	10 item measure of mindfulness skills. Normed on four samples of school age children and adolescents. Response options range from (0) *never true* to (4) *always true.*	Correlated with quality of life, school and social functioning and mindfulness-inconsistent processes (e.g., externalizing behavior).
Avoidance and Fusion Questionnaire for Youth (AFQ-Y; [57]) *	17 item measure of psychological inflexibility for youth. There is also a validated eight-item short form of the AFQ-Y.	Scores for both versions correlated with child anxiety, somatic complaints, mindfulness, quality of life, and scholastic functioning.
**Parent specific**		
Parent psychological flexibility measure (PPFQ; [43,62])	24 item measure to assess parental levels of psychological flexibility in responses to child’s pain symptoms. Items range from (0) *never true* to (6) *always true.*	Scores correlated with child disability, depression, and pain acceptance, as well as with parental response behaviors, as assessed by the Adult Responses to Child Symptoms (ARCS) measure [63].
Chronic Pain Acceptance Questionnaire: Parent report (CPAQ-P; [64])	16 item self-report measure of parent perceptions of child’s acceptance of pain, adapted from the adult CPAQ [33]. Same two subscales as the CPAQ-A. Response options range from (0) *never true* to (6) *always true.*	Scores correlated with child pain intensity and disability, as well as parent pain catastrophizing, pain-related fear, and maladaptive protective responses.
Parent Pain Acceptance Questionnaire (PPAQ; [42])	15 item self-report measure of parent’s own levels of acceptance towards their child’s pain. Adapted from the CPAQ-P [64]. Two sub-scales: (1) Activity Engagement and (2) Pain-related Thoughts and Feelings.	Scores correlated with child pain acceptance, pain-related fear, and pain catastrophizing, as well as parental maladaptive responses to pain and pain catastrophizing
Parental Acceptance and Action Questionnaire (PAAQ [59]) *	15 item self-report measure of experiential avoidance in relation to parenting. Two subscales: (1) Inaction and (2) Unwillingness	In preliminary validation, the PAAQ correlated with symptoms of child psychopathology and measures of controlling parental behaviors and affective expression. Predicted significant amounts of variance in parent and clinician ratings of child anxiety symptoms.

* Not a pain specific ACT measure.
